# Sexual experiences, behaviours and attitudes in the parents in the Avon Longitudinal Study of Parents and Childhood (ALSPAC)

**DOI:** 10.12688/wellcomeopenres.21263.1

**Published:** 2024-05-09

**Authors:** Yasmin Iles-Caven, Jean Golding

**Affiliations:** 1 Population Health Sciences, Bristol Medical School, University of Bristol, Bristol, England, BS8 2BN, UK

**Keywords:** ALSPAC, Early sexual experiences, sexual abuse, sexual debut, parents, sexual orientation, sexual functioning

## Abstract

The aim of this data note is to describe the sexual experiences, behaviours, and attitudes data collected from the parent cohorts of the Avon Longitudinal Study of Parents and Children (ALSPAC) over a 30-year period. ALSPAC is an ongoing birth cohort which enrolled 14,541 pregnant women living in Avon, a county of southwest England, with expected dates of delivery between April 1991 and December 1992 inclusive and continues collecting data on these mothers (age range at delivery <16–41 years), their partners (age range at delivery <16–65), and their offspring, so far resulting in 100,000+ phenotype variables.

During the index pregnancy the mothers were asked (and at 8 months post-delivery for partners) about their early sexual experiences to identify sexual abuse/assaults and the age at which an event first happened. Both parents were asked also about sexual abuse within a battery of questions identifying Adverse Childhood Events (ACE). Further longitudinal data described here includes satisfaction with sexual and non-sexual sides of their relationship; sexual functioning; and at seven years post-delivery, both parents were asked to describe their sexual orientation as well as the sex of their partners and cohabiting partners.

These data provide the ability to compare generational differences between parental sexual behaviours and attitudes with those of their offspring, as well as allowing comparisons with other longitudinal surveys where similar (or identical) information has been collected such as the National Survey of Sexual Attitudes and Lifestyles (NATSAL-3).

This paper forms a companion to another data note describing similar data collected on the offspring cohort.

## Background

This paper describes data that has been collected over time on sexual experiences, behaviours and attitudes in the Avon Longitudinal Study of Parents and Children (ALSPAC). Data on early sexual experiences were initially collected retrospectively from the parent cohorts during the index pregnancy (1990–1992) for the mothers and at 8 months post-birth for their partners. Later data sweeps have included identical questions to that of the National Survey of Sexual Attitudes & Lifestyles (NATSAL) which has undertaken four national surveys in Britain ( About | Natsal). NATSAL used a probability sampling method to randomly select people across Britain who were broadly representative of the general population and could be used to look at differences in sexual behaviours over time. Although undertaken by face-to-face interviews, sensitive topics were self-completed directly onto a computer (CADI). In NATSAL3, over 15,000 respondents born between the 1930s and 1990s were interviewed between 2010 and 2012: and can be compared with ALSPAC (mothers born 1950 – 1974, their partners (born 1926–1975)) and their offspring born 1991–1992 (see Table 1 for age of the parents at the time of the index birth).

**Table 1.** Basic sociodemographic description of the ALSPAC parent cohorts during the index pregnancy (variables used are in italics, mother/partner).

Sociodemographic description	Mothers N	%	Partners N	%
**Number of older ** **brothers** *c450/pf2080*				
None	7343	59.0	1566	42.5
1+	5096	41.0	2122	57.5
**Number of older sisters** *c451/pf2081*				
None	7562	60.9	1642	45.1
1+	4870	39.2	1997	54.9
**Number of younger** ** brothers** *c453/pf2082*				
None	7690	61.9	1631	53.6
1+	4742	38.1	2113	56.4
**Number of younger ** **sisters** *c454/pf2083*				
None	7806	62.8	1685	45.2
1+	4628	37.2	2040	54.8
**Was a twin** *c456/pf2084*	247	2.0	126	2.4
**Age at delivery (in** **1991/92)** *mz028b;* * partner_age*				
<25	4676	30.7	1832	15.2
25–34	9153	60.2	7714	63.8
35+	1378	9.1	2539	21.0
Total	15207		12085	
*Range (years)*	*<16–41*		*<16–65*	
**Ethnicity** *c800, c801*				
White	11902	97.4	11493	96.0
Non-white	320	2.6	476	4.0
Total	12222		11969	
**Highest educational ** **attainment** *c645a, * *pb325a*				
≤0 level	7969	64.7	5143	52.6
A level+	4344	35.3	4639	47.4
Total	12313		9782	
**Marital status** *a525 * *pa065*				
Currently married	9996	74.8	6932	81.8
Not currently married	3369	25.2	1541	18.2
Total	13365		8473	
**Social class ** *c755, c765*				
Non-manual (I-IIINM)	7990	80.1	6066	55.6
Manual (IIIM-V)	1983	19.9	4787	44.1
Total	9973		10853	
**Housing tenure** *a006*			-	-
Owner occupied	9732	73.1		
Rented/all other	3577	26.9		
Total	13309			
**Urban/Rural** *Jan1993ur01ind_m*			-	-
Urban	11676	90.2		
Town & fringe	528	4.1		
Village	531	4.1		
Hamlet/isolated dwelling	202	1.6		
Total	12937			
**IMD** *Jan2014md2010q5*			-	-
1 least deprived	3936	30.5		
2	2939	22.8		
3	2281	17.7		
4	2108	16.3		
5 most deprived	1631	12.6		
Total	12895			

## Methods

### Participants

***The Avon Longitudinal Study of Parents and Children (ALSPAC).*** All pregnant women (G0 mothers) resident in the Avon area of south-west England with expected dates of delivery between April 1991 and December 1992 inclusive were invited to enrol in ALSPAC. The initial number of pregnancies enrolled was 14,541, resulting in 13,988 infants who were alive at age 12 months. Additional phases of enrolment are described in more detail in the cohort profile papers ( Boyd *et al.*, 2013; Fraser *et al.*, 2013; Major-Smith *et al.*, 2023; Northstone *et al.*, 2023). The total sample size for analyses using any data collected after the age of seven is 15,447 pregnancies, resulting in 15,658 foetuses. Of these, 14,901 children (G1 cohort) were alive at 1 year of age. Some women enrolled with one or more pregnancies, consequently the number of unique mothers who were enrolled was 14,833 in total. G0 partners were invited to complete questionnaires by the mothers at the start of the study but they were not formally enrolled at that time. Note that ‘partners’ were often the biological father (at least around initial enrolment), but subsequently may be a stepfather, father-figure, or same-sex partner. 12,113 G0 partners have been in contact with the study by providing data and/or formally enrolling when this commenced in 2010. 3,807 G0 partners (mean age 63 years in 2023) are currently enrolled (via the mother or the study child) ( Northstone *et al.*, 2023), and may not necessarily still be with the study mother. Since 2014, study data are collected and managed using REDCap electronic data capture tools hosted at the University of Bristol. REDCap (Research Electronic Data Capture) is a secure, web-based software platform designed to support data capture for research studies ( Harris *et al.*, 2009). For those participants who prefer to complete paper versions these are also offered. Completed paper questionnaires are scanned into electronic data using Teleform data capture software. Please note that the study website contains details of all the data that is available through a fully searchable data dictionary and variable search tool: http://www.bristol.ac.uk/alspac/researchers/our-data/.

Ethical approval for the ALSPAC study was obtained from the ALSPAC Ethics and Law Committee (ALEC; IRB00003312) and the Local Research Ethics Committees. Detailed information on the ways in which confidentiality of the cohort is maintained may be found on the study website: http://www.bristol.ac.uk/alspac/researchers/research-ethics/. All methods were performed in accordance with the relevant guidelines and regulations. Informed consent for the use of data collected via questionnaires and clinics was assumed from participants following the recommendations of the ALSPAC Ethics and Law Committee at the time ( Birmingham, 2018).

### Data collection and frequencies

Data on sexual experiences, attitudes and behaviours have been collected over time from the both the parent (G0) and offspring (G1) cohorts. The main focus of this data note is to describe the data collected from the parents relating to early sexual experiences; satisfaction with the affection/sexual side of their relationships; sexual functioning and sexual identity.

Basic demographics of the parents during pregnancy are presented in Table 1.

Satisfaction with sexual relationships and frequency and enjoyment of intercourse have been asked of the mothers and their partners over time. Frequency of sexual intercourse was asked of the mothers annually for the first 6 years post-delivery, and thereafter at 9, 12 and 19 years after the index birth. The mothers’ frequencies remained fairly stable for all questions over time, but it should be noted that she may have changed partners between follow-ups. Change of partner data has recently been added to the main database ( Northstone *et al.*, 2023). Perhaps not surprisingly, those reporting not having sexual intercourse at all increased steadily from 8.7% at ~2 years post-delivery to 19.6% 17 years later; those having intercourse at least once per week remained at over 50% until 12 years post-delivery (47.5%) and dropped to 40.9% by 19 years post-delivery ( Table 2a). Interested researchers may also wish to compare the data reported here with the quality of relationships which used the Intimate Bond Measure (see Tohidinik *et al.*, 2023).

**Table 2a.** Satisfaction with sexual relationship and frequency and enjoyment of intercourse over time – **Mothers**.

Age of Index Child at time of enquiry	1yr 9mth	2yr 9mth	3yr 11mth	5yr 1mth	6yr 1mth	9yr 2mth	12yr 1mth	19yr 2010
*Frequency respondent having sex nowadays (variable/N)*	*G130 10116*	*H095 9446*	*J110 9301*	*K1190 8676*	*L3270 8338*	*P1230 7656*	*S1230 6705*	*T4500 3950*
Not at all	902 (8.7)	938 (9.9)	861 (9.3)	838 (9.7)	808 (9.7)	828 (10.8)	886 (13.2)	775 (19.6)
<Once/month	1069 (10.6)	1112 (11.8)	997 (10.7)	883 (10.2)	974 (11.7)	946 (12.4)	913 (13.6)	698 (17.7)
1–3 times/month	2734 (27.0)	2512 (26.6)	2467 (26.5)	2264 (26.1)	2084 (25.0)	1928 (25.2)	1726 (25.7)	860 (21.8)
About once/week	2599 (25.7)	2389 (25.3)	2358 (25.4)	2147 (24.7)	2033 (24.4)	1880 (24.6)	1507 (22.5)	860 (21.8)
2–4 times/week	2588 (25.6)	2291 (24.3)	2392 (25.7)	2351 (27.1)	2237 (26.8)	1914 (25.0)	1533 (22.9)	705 (17.8)
>5 times/week	224 (2.2)	204 (2.2)	226 (2.4)	193 (2.2)	202 (2.4)	160 (2.1)	140 (2.1)	52 (1.3)
*Is this as often as prior to index pregnancy (variable/N)*	*G131 9990*	*-*	*-*	*-*	*-*	*-*	*-*	*-*
More often	561 (5.6)							
About as often	4137 (41.4)							
Less often	5292 (53.0)							
*Satisfaction level with relationship – affection now* *(variable/N)*	*G695 10107*	*H566 9463*	*-*	*-*	*L6131 7756*	*P3131 7092*	*S3131 6125*	*-*
Very satisfied	4311 (45.0)	3757 (42.4)			3306 (42.6)	2993 (42.2)	2640 (43.1)	
Satisfied	4246 (44.3)	3811 (43.0)			3170 (40.9)	2906 (41.0)	2433 (39.7)	
Dissatisfied	847 (8.8)	1039 (11.7)			980 (12.6)	912 (12.9)	802 (13.1)	
Very dissatisfied	171 (1.8)	247 (2.8)			300 (3.9)	281 (4.0)	250 (4.1)	
No partner	548	609	664	707	575	621	642	710
*Satisfaction level with relationship – sex now (variable/N)*	*G697 10089*	*H567 9423*	*-*	*-*	*L6132 7717*	*P3132 7020*	*S3132 6025*	*-*
Very satisfied	3844 (40.3)	3592 (40.8)			3312 (42.9)	2844 (40.5)	2464 (40.9)	
Moderately satisfied	4088 (42.8)	3784 (42.9)			3106 (40.2)	2931 (41.8)	2501 (41.5)	
Dissatisfied	1244 (13.0)	1105 (12.5)			951 (12.3)	898 (12.8)	759 (12.6)	
Very dissatisfied	365 (3.8)	333 (3.8)			348 (4.5)	347 (4.9)	301 (5.0)	
No partner	548	609	664	707	575	621	642	710
*Extent respondent enjoys sex (variable/N)*	*-*	*H096 9348*	*J111 9170*	*K1191 8585*	*L3271 8213*	*P1231 7548*	*S1231 6570*	*T4501 3870*
Yes, very much	-	4255 (45.5)	4198 (45.8)	4020 (46.8)	3859 (47.0)	3539 (46.9)	2926 (44.5)	1536 (39.7)
Yes, somewhat	-	3574 (38.2)	3565 (38.9)	3290 (38.3)	3161 (38.5)	2861 (37.9)	2551 (38.8)	1411 (36.5)
No, not a lot	-	791 (8.5)	695 (7.6)	615 (7.2)	589 (7.2)	524 (6.9)	423 (6.4)	288 (7.4)
No, not at all	-	68 (0.7)	62 (0.7)	63 (0.7)	78 (0.9)	54 (0.7)	42 (0.6)	44 (1.1)
No sex currently	-	660 (7.1)	650 (7.1)	597 (7.0)	526 (6.4)	570 (7.6)	628 (9.6)	591 (15.3)

No partner totals from 3yrs 11mth onwards uses variables: J600; K8024; L6000; P3000; S3000; T1065.

At 8 weeks post-delivery, the partners were asked about their attraction to the mother and satisfaction with their current sex lives. The majority (80.5%) never felt unattracted to the mother since the birth and almost half were usually happy with the sexual side of the relationship, but 10.6% never felt happy about it. Over 55% reported less sexual intercourse at 21 months post-delivery than prior to the pregnancy. As noted with the mothers, partner’s frequencies remained fairly stable between 21 months and 12 years after the birth ( Table 2b). Between these time points at least 65% of partners reported they very much enjoyed sex compared with 39.7% of women ( Table 2a and Table 2b).

**Table 2b.** Satisfaction with sexual relationship and frequency and enjoyment of intercourse over time – **Partners** (<5 may equal zero).

Age of Index Child at time of enquiry	8 weeks	1yr 9mth	2yr 9mth	3yr 11mth	5yr 1mth	6yr 1mth	9yr 2mth	12yr 1mth
*Since the birth, I have not felt attracted to my partner * *(variable/N)*	*PC320 * *8221*	*-*	*-*	*-*	*-*		*-*	*-*
Usually feel this way	136 (1.7)							
Often feel this way	158 (1.9)							
Sometimes feel this way	1310 (15.9)							
Never feel this way	6617 (80.5)							
*I cannot bear to be touched by my partner (variable/N)*	*PC321 * *8228*	*-*	*-*	*-*	*-*		*-*	*-*
Usually feel this way	21 (0.3)							
Often feel this way	24 (0.3)							
Sometimes feel this way	230 (2.8)							
Never feel this way	7953 (96.7)							
*I’m happy with our sexual relationship (variable/N)*	*Pc322 * *8207*	*-*	*-*	*-*	*-*		*-*	*-*
Usually feel this way	3932 (47.9)							
Often feel this way	1621 (19.8)							
Sometimes feel this way	1780 (21.7)							
Never feel this way	874 (10.6)							
*Frequency respondent having sex nowadays (variable/N)*	*-*	*Pe140 * *6057*	*Pf1160 * *5377*	*Pg1160 * *5031*	*Ph1190 * *4404*	*Pj3270 * *4400*	*Pm1230 * *3600*	*Pq1230 * *3209*
Not at all		405 (6.7)	394 (7.3)	317 (6.3)	264 (6.0)	265 (6.0)	240 (6.7)	268 (8.4)
<Once/month		829 (13.7)	772 (14.4)	662 (13.2)	559 (12.7)	605 (13.8)	565 (15.7)	565 (17.6)
1–3 times/month		1815 (30.0)	1656 (30.8)	1575 (31.3)	1399 (31.8)	1324 (30.1)	1095 (30.4)	1005 (31.3)
About once/week		1532 (25.3)	1303 (24.2)	1216 (24.2)	1043 (23.7)	1074 (24.4)	877 (24.4)	667 (20.8)
2–4 times/week		1372 (22.7)	1156 (21.5)	1168 (23.2)	1061 (24.1)	1055 (24.0)	766 (21.3)	653 (20.3)
>5 times/week		104 (1.7)	96 (1.8)	93 (1.8)	78 (1.8)	77 (1.8)	57 (1.6)	51 (1.6)
*Is this as often as prior to index pregnancy (variable/N)*	*-*	*Pe141 * *6037*	*-*	*-*	*-*	*-*	*-*	
More often		222 (3.7)						
About as often		2476 (41.0)						
Less often		3339 (55.3)						
*Satisfaction level with relationship – affection now * * (variable/N)*	*-*	*Pe371 * *6085*	*Pf6041 * *5356*	*-*	*-*	*Pj6131 * *4359*	*Pm3131 * *3519*	*-*
Very satisfied		2432 (40.0)	2068 (38.6)			1634 (37.5)	1318 (37.5)	
Moderately satisfied		2739 (45.0)	2376 (44.4)			1937 (44.4)	1569 (44.6)	
Somewhat dissatisfied		752 (12.4)	735 (13.7)			636 (14.6)	497 (14.1)	
Very dissatisfied		162 (2.7)	177 (3.3)			152 (3.5)	135 (3.8)	
No partner		-	-			-	-	
*Satisfaction level with relationship – sex now (variable/N)*	*-*	*Pe372 * *6090*	*Pf6042 * *5327*	*-*	*-*	*Pj6132 * *4353*	*Pm3132 * *3508*	*-*
Very satisfied		1852 (30.4)	1682 (31.6)			1454 (33.4)	1145 (32.6)	
Moderately satisfied		2432 (39.9)	2213 (41.5)			1771 (40.7)	1413 (40.3)	
Somewhat dissatisfied		1307 (21.5)	1066 (20.0)			814 (18.7)	649 (18.5)	
Very dissatisfied		499 (8.2)	366 (6.9)			314 (7.2)	301 (8.6)	
No partner		-	-			-	-	
*Extent respondent enjoys sex (variable/N)*	*-*	*-*	*Pf1161 * *5361*	*Pg1161 * *4998*	*Ph1191 * *4374*	*Pj3271 * *4376*	*Pm1231 * *3573*	*Pq1231 * *3161*
Yes, very much			3616 (67.5)	3422 (68.5)	2987 (68.3)	2996 (68.5)	2456 (68.7)	2084 (65.9)
Yes, somewhat			1342 (25.0)	1258 (25.2)	1089 (24.9)	1114 (25.5)	872 (24.4)	800 (25.3)
No, not a lot			107 (2.0)	81 (1.6)	81 (1.9)	71 (1.6)	64 (1.8)	64 (2.0)
No, not at all			5 (0.1)	<5	<5	<5	<5	<5
No sex currently			291 (5.4)	235 (4.7)	214 (4.9)	191 (4.4)	179 (5.0)	209 (6.6)

In 2013, maternal data (Vfiles) were collected on concerns about current sex drive/desire and sexual functioning via a self-completion questionnaire. Similar questions were asked of her partner at the Focus on Fathers clinic (n=2024) (FPA files, data collected between 2011and 2013). For those fathers who did not attend the clinic, postal questionnaires were administered (n=483). Additional questions on erectile dysfunction of the partner were also asked of both parents ( Table 3). All questions administered to the partners comprised the European Male Ageing Study Sexual Function Questionnaire EMAS-SFQ ( O’Connor *et al.*, 2008). Table 3 shows that the majority of mothers (80.3%) and partners (91.9%) lived with their sexual partner/spouse. The frequency with which the mothers versus the partners thought about sex (with eight possible responses) is interesting with much higher proportions of partners thinking about sex more often: 11.8% of mothers vs 0.5% of partners never thinking about sex at all; once a week 15% and 4.4%; 2–3 times per week 22.1% and 15.5% respectively. From 4–6 times/week double the partners thought about sex; with the largest gap being more than once per day 5.2% for the mothers vs. 39.7% for their partners.

**Table 3.** Mother and Partner’s sexual functioning frequencies (mother’s data collected in 2013 via ‘Your life in 2013’ questionnaire; partner’s data collected at Focus on Fathers clinic 2011–13 via an online questionnaire and, for those not attending, a paper self-completion questionnaire at home. [- not asked] (<5 may equal zero).

	Mothers 2013	Partners 2011–2013
*Respondent’s sexual partner* * circumstances are best described* * as*	*v5000*	*fpa7050*
Lives with spouse	N/A	1819 (85.4)
Lives with sexual partner	3716 (80.3)	139 (6.5)
Has sexual partner but do not cohabit	325 (7.0)	87 (4.1)
No sexual partner	586 (12.7)	84 (3.9)
	4627	2129
*Frequency respondent thinks* * about sex*	*v5001*	*fpa7100*
Not at all	526 (11.8)	10 (0.5)
Once in previous month	520 (11.7)	17 (0.8)
2–3 times in previous month	955 (21.5)	50 (2.4)
Once a week	670 (15.0)	92 (4.4)
2–3 times per week	984 (22.1)	324 (15.5)
4–6 times per week	359 (8.1)	354 (16.9)
Once a day	206 (4.6)	415 (19.8)
More than once a day	232 (5.2)	832 (39.7)
	4452	2094
*Respondent worried by current * *level of sex drive/desire*	*v5002*	*fpa7101*
Not worried at all	3213 (71.2)	1532 (72.6)
A little worried	917 (20.3)	381 (18.1)
Moderately worried	317 (7.0)	139 (6.6)
Very worried	58 (1.3)	41 (1.9)
Extremely worried	10 (0.2)	17 (0.8)
	4515	2110
		
*Respondent’s sex drive/desire has * *changed compared with a year * *ago*	*v5003*	*fpa7102*
Increased a lot	93 (2.1)	26 (1.2)
Increased moderately	229 (5.1)	112 (5.3)
Neither increased nor decreased	2668 (59.3)	1471 (69.7)
Decreased moderately	1001 (22.2)	439 (20.8)
Decreased a lot	511 (11.4)	62 (2.9)
	4502	2110
*Mother had a sexual partner* * within previous month*	*v5004*	*-*
Yes	3329 (74.4)	
No	1143 (25.6)	
	4472	
*No. times respondent attempted* *sexual intercourse within previous* * month*	*v5005*	*fpa7103*
Not at all	743 (19.9)	226 (12.2)
Once in previous month	516 (13.8)	227 (12.2)
2-3 times in previous month	978 (26.1)	444 (23.9)
Once a week	652 (17.4)	469 (25.3)
2-3 times per week	695 (18.6)	397 (21.4)
4-6 times per week	131 (3.5)	74 (4.0)
Once a day	13 (0.3)	12 (0.6)
More than once a day	12 (0.3)	6 (0.3)
	3740	1855
*Frequency respondent engages* *in sexual activities but not* * intercourse*	*v5006*	*fpa7104*
Not at all	827 (22.4)	218 (11.7)
Once in previous month	269 (7.3)	105 (5.6)
2-3 times in previous month	431 (11.7)	286 (15.4)
Once a week	317 (8.6)	156 (8.4)
2-3 times per week	624 (16.9)	351 (18.9)
4–6 times per week	332 (9.0)	214 (11.5)
Once a day	399 (10.8)	248 (13.3)
More than once a day	490 (13.3)	284 (15.3)
	3689	1862
*Frequency respondent* * masturbates*	v5007	fpa7105
Not at all	2332 (55.0)	454 (23.2)
Once in previous month	866 (20.4)	256 (13.1)
2–3 times in previous month	575 (13.6)	272 (13.9)
Once a week	245 (5.8)	398 (20.4)
2–3 times per week	183 (4.3)	386 (19.8)
4–6 times per week	25 (0.6)	123 (6.3)
Once a day	9 (0.2)	54 (2.8)
More than once a day	<5	10 (0.5)
	4239	1953
*Respondent worried by frequency * *of sexual activities*	*v5008*	*fpa7106*
Not at all worried	3130 (71.7)	1324 (63.6)
A little worried	821 (18.8)	497 (23.9)
Moderately worried	335 (7.7)	189 (9.1)
Very worried	64 (1.5)	51 (2.4)
Extremely worried	17 (0.4)	22 (1.1)
If worried by frequency:	4367	2083
*Respondent considers frequency* * of sexual activities to be*	*v5009*	*fpa7107*
Too frequent	31 (2.6)	27 (3.6)
Not frequent enough	1150 (97.4)	714 (96.4)
	1181	741
*Respondent’s sex activities have* * changed compared with a year * *ago*	*v5010*	*fpa7108*
Increased a lot	89 (2.0)	26 (1.3)
Increased moderately	196 (4.5)	98 (4.7)
Neither increased nor decreased	2629 (60.1)	1294 (62.6)
Decreased moderately	1003 (22.9)	554 (26.8)
Decreased a lot	460 (10.5)	95 (4.6)
	4377	2067
*Frequency in previous month that * *partner was able to get/keep an * *erection*	*v5011*	*fpa7109*
Always	1880 (43.9)	1365 (65.8)
Usually	533 (12.5)	490 (23.6)
Sometimes	349 (8.2)	175 (8.4)
Never	638 (14.9)	45 (2.2)
Not applicable	879 (20.5)	-
	4279	2075
*Partner worried/distressed by * *current ability to have erection*	*-*	*fpa7110*
Not at all		1580 (76.6)
A little worried		317 (15.4)
Moderately worried		121 (5.9)
Very worried		38 (1.8)
Extremely worried		7 (0.3)
		2063
*Partner’s ability to have erection* * changed compared with a year * *ago*	*-*	*fpa7111*
Increased a lot		8 (0.4)
Increased moderately		39 (1.9)
Neither increased nor decreased		1711 (82.5)
Decreased moderately		287 (13.8)
Decreased a lot		30 (1.4)
		2075
*Frequency of feeling orgasm/* *climax when sexually stimulated*	*v5012*	*fpa7112*
Almost never or never	258 (6.0)	20 (1.0)
Few times (< than half the time)	351 (8.2)	50 (2.5)
Sometimes (about half the time)	730 (17.1)	91 (4.5)
Most times (> than half the time)	691 (16.2)	379 (18.6)
Almost always or always	1931 (45.2)	1466 (71.9)
No intercourse or masturbation	309 (7.2)	32 (1.6)
	4270	2038
*Respondent worried by current * *orgasmic experience*	*v5013*	*fpa7113*
Not at all worried	3699 (86.9)	1705 (83.5)
A little worried	378 (8.9)	230 (11.3)
Moderately worried	147 (3.5)	90 (4.4)
Very worried	24 (0.6)	14 (0.7)
Extremely worried	7 (0.2)	<5
	4255	2043
*Respondent’s enjoyment of * *orgasm experience has changed * *compared with a year ago*	*v5014*	*fpa7114*
Increased a lot	76 (1.8)	21 (1.0)
Increased moderately	181 (4.3)	87 (4.2)
Neither increased nor decreased	3286 (78.6)	1695 (82.7)
Decreased moderately	470 (11.2)	218 (10.6)
Decreased a lot	169 (4.0)	29 (1.4)
*Frequency partner awoke with full * *erection*	*-*	*fpa7115*
Not at all		295 (14.3)
Once in past month		272 (13.2)
2–3 times in past month		382 (18.6)
Once a week		329 (16.0)
2–3 times a week		481 (23.4)
4–6 times a week		184 (8.9)
Once a day		105 (5.1)
More than once a day		10 (0.5)
		2058
*Partner worried by frequency of * *morning erections*	*-*	*fpa7116*
Not at all		1877 (91.5)
A little worried		105 (5.1)
Moderately worried		55 (2.7)
Very worried		12 (0.6)
Extremely worried		<5
		2051
*Frequency of morning erections* * changed compared with a year * *ago*	*-*	*fpa7117*
Increased a lot		5 (0.2)
Increased moderately		36 (1.8)
Neither increased nor decreased		1692 (82.7)
Decreased moderately		287 (14.0)
Decreased a lot		25 (1.2)
		2045
*Degree to which respondent is* * satisfied with overall sex life*	*v5015*	*fpa7118*
Very satisfied	1338 (31.4)	424 (20.5)
Moderately satisfied	952 (22.3)	483 (23.4)
About equally satisfied and dissatisfied	901 (21.1)	355 (17.2)
Moderately dissatisfied	709 (16.6)	483 (23.4)
Very dissatisfied	366 (8.6)	243 (11.8)
	4266	2065
*Degree to which respondent is * *satisfied with relationship with* * partner (non-sexual)*	*v5016*	*fpa7119*
Very satisfied	2196 (51.2)	1099 (54.4)
Moderately satisfied	796 (18.5)	436 (21.6)
About equally satisfied and dissatisfied	481 (11.2)	214 (10.6)
Moderately dissatisfied	269 (6.3)	145 (7.2)
Very dissatisfied	146 (3.4)	126 (6.2)
No partner	405 (9.4)	n/a
	4293	2020

The majority of mothers and partners had no concerns at all about their current sex drive (over 70% for both) and when asked if their sex drive had changed over the last 12 months, the majority (59.3% mothers and 69.7% partners) reported it was about the same, the largest difference being for the ‘decreased a lot’ groups at 11.4% for mothers and 2.9% for partners ( Table 3).

Partners were generally slightly more likely to attempt sexual intercourse more often than the mothers, but 19.9% of mothers and 12.2% of partners had not tried at all during the previous month. Frequencies for once/day or more than once/day were very similar between them, but small ( Table 3). Masturbation in the previous month was also more frequent in men: 55% of mothers vs. 23.2% of partners had not masturbated at all, compared with once per week 5.8% vs. 20.4% respectively; and once per day 0.2% vs. 2.8% respectively.

The ability of the partner to get and maintain an erection was asked of the mothers and of the enrolled partners ( Table 3) 20–22 years post-delivery. Always able to get an erection was stated by 65.8% of partners but 43.9% of mothers and ‘never’ was reported by 2.2% of partners vs. 14.9% of mothers – this may be due to a number of the enrolled partners (who were usually the biological father) not being the current partner of the mother, although partner changes over time since the pregnancy have been ascertained to be <4% (with a further 2.1% undeterminable) ( Northstone *et al.*, 2023).

Only the partners were asked if they were worried about their ability to have an erection and the majority were unconcerned (76.6%), but 2.1% claimed to be very or extremely worried; over 15% stated that their ability had decreased over the past year ( Table 3). 23.4% of partners reported waking with a full erection 2–3 times/week and 8.9% at 4–6 times/week and ‘never’ 14.3%. The vast majority (82.7%) said there had been no change in morning erections within the previous year, but over 15% reported a large decrease ( Table 3).

Over half the mothers and partners reported being very satisfied with their (non-sexual) relationship in general, but 9.7% of mothers vs. 13.4% of partners reported being dissatisfied. Compare these figures to satisfaction with the sexual side of the relationship, where 31.4% of women said they were very satisfied compared with 20.5% of partners; for those who were dissatisfied with the sexual side, the frequencies are 25.2% and 34.2% respectively ( Table 3).

One paper using these data ( Martin *et al.*, 2022) looked at mode of delivery and maternal sexual well-being at four time points between 33 months and 18 years post-partum and sex-related pain (vaginal during sex and elsewhere post-coitus) at 11 years post-partum. They found no association between mode of delivery and sexual frequency or enjoyment at 33 months, but on adjustment, Caesarean section was associated with painful sex compared with a vaginal delivery 11 years after the birth.

Questions eliciting Adverse Childhood Events (ACEs) had been asked of the G0 cohorts during the index pregnancy. The following papers describe these in detail, including sexual assault or child sexual abuse:

*The coding of text in a life events diary completed by the mothers in an antenatal questionnaire (Dfiles) – this includes sexual abuse, sexual assault and rape ( Gregory *et al.*, 2023).

* ACEs in ancestors were described in an interview study ( Birmingham *et al.*, 2022) with some qualitative reports of sexual violence/abuse to parents and grandparents of the G0 parents.

*ACEs in the parents (G0s) where 5.1% of mothers and 1.2% of partners reported sexual abuse (variables maternal c422a; partner pb472a) and also the subjective effect of such events ( Ellis *et al.*, 2020).

All questions on early sexual experiences are listed in the *Extended data* (Supplementary Table 1 ( Iles-Caven, 2024)) along with the variable names. These comprise:

The questionnaires administered to the mothers at ~32 weeks gestation (Cfiles) and to her partner at 8 months after the birth (PDfiles), contained a Life Events scale which included the question ‘Were you sexually abused before the age of 17?’, with yes/no/not sure options, followed by ‘If yes, did this affect you a lot, moderately, mildly, yes but no effect at all?’ The life events questions were administered at intervals thereafter to cover the period since the previous enquiry. Frequencies for these questions can be found in Table 4. Mothers reported more sexual abuse than partners (5.1% vs. 1.2%) and were also more likely to feel that the abuse had affected them a lot (3.0% vs. 0.5%). The mothers had much higher frequencies of sexual assault up to the point of enquiry in pregnancy than partners (4.1% vs. 0.9%), an additional 1.2% of mothers were assaulted within the first 11 years of their child’s life.

**Table 4.** Self-reported sexual abuse and sexual assaults experienced by mothers and partners (<5 may contain zero).

Sexual Abuse/Assaults	Mother	Partner
*Life events: Sexually abused <17 yrs*	*c422a*	*pb472a*
Yes	631 (5.1)	124 (1.2)
No	11681 (94.9)	9876 (98.8)
*Degree of effect of sexual abuse*	*c422*	*pb472*
Yes, affected me a lot	370 (3.0)	53 (0.5)
Yes, moderate	131 (1.1)	24 (0.2)
Yes, mild	94 (0.8)	23 (0.2)
Yes, happened but had no effect	36 (0.3)	24 (0.2)
Did not happen	11681 (94.9)	9876 (98.8)
*Sexually assaulted ever*	*d102*	*pa132*
Yes	497 (4.1)	79 (0.9)
No	11690 (95.9)	8318 (99.1)
Age range (Yrs)	2–34	1–33
*Sexually assaulted within 7 years * *since baby’s birth*	*m1029*	*pk1029*
Yes	66 (0.8)	<5
No	8213 (99.2)	3989 (100.0)
*Sexually assaulted within last * *4 years (when index child aged * *11 years)*	*r3529* *7531*	*pp3529* *3614*
Yes	29 (0.4)	<5
No	7502 (99.6)	3611 (99.9)

The final section of these early questionnaires comprised a set of seven questions used to identify any childhood sexual abuse. They were developed by Dr. Jean Price and the ALSPAC team after a review of the literature which stressed the importance of asking specific questions concerning actual events rather than the more general ‘have you been sexually abused?’ Advice was sought from the pregnant G0 women via a committee formed specifically to advise on this, along with extensive piloting and considerable discussion with the ALSPAC Ethics & Law Committee (ALEC). 

It should be noted that mothers under the age of 16 along with those mothers who had also enrolled subsequent pregnancies into the study (and therefore answered the questions previously) had a questionnaire which omitted the early sexual experiences questions on advice from ALEC (n=135). 

The section was preceded with a page with the following statement:

“These next pages are concerned with early sexual experiences. IF YOU WOULD RATHER NOT ANSWER THEM, WE QUITE UNDERSTAND. JUST STOP NOW AND SEND THE QUESTIONNAIRE BACK AS USUAL. But it is possible that whether or not such events had taken place they may be a vital clue in understanding some of the problems we are trying to solve – even though they may appear unconnected. If you feel you can help, we would be very grateful.”

Despite this warning the section was only omitted by 10% of the mothers. On turning the page, the leading paragraph stated:

“As we are growing up, we all have sexual experiences. These are a normal part of development and learning. Some people have unwanted experiences to which they do not agree. These experiences can be important and may affect how you feel about yourself, your partner and your baby. Below are questions which ask about your sexual experiences from childhood until the present time”.

1. Did anyone ever purposely expose/flash themselves to you before you were 16?
2. Did anyone masturbate in front of you before you were 16?
3. Did anyone ever touch or fondle your body, including your breasts or genitals, or attempt to arouse you sexually before you were 16?
4. Did anyone try to have you arouse them, or touch their body in a sexual way before you were 16?
5. Did anyone rub their genitals against your body in a sexual way before you were 16?
6. Did anyone have sexual intercourse with you before you were 16?
7. Did anyone try to put their penis into your mouth before you were 16?

For each of these questions, three options were given:

a. Yes, happened once only.
b. Yes, happened more than once.
c. No, did not happen.

Each of the questions then had a follow-on question, If yes,

(i). Who was involved? (No, yes).
(ii). Did you want this to happen with this person? (No, yes, unsure).”

These questions were asked for the following perpetrators separately: boyfriend, girlfriend (except for situations where a female was not feasible), parent or parent figure, brother or sister, other relatives, family friends, stranger, other person (please describe). 

Table 5 gives frequencies for each of the seven sexual experiences listed above for the total number of positive responses and if the event happened more than once. Being flashed at occurred more often to the mothers than partners (26.5% vs. 11.6%), as did ‘someone tried to put their penis in your mouth’ (8.5% vs. 1.2%). However, frequencies of sexual intercourse prior to age 16 were similar for both groups (20.0% and 20.8% respectively).

**Table 5.** Sexual experiences prior to age 16 in the mothers and their partners (C and Pd files).

	Mothers	N=10,977	Partners	N=6,437
Event	All Yes N (%)	>1 times N (%)	All Yes N (%)	>1 times N (%)
Someone purposely flashed/exposed themselves to you	2906 (26.5)	1064 (9.7)	748 (11.6)	453 (7.0)
Someone masturbated in front of you	936 (8.6)	508 (4.7)	559 (8.7)	342 (5.3)
Someone touched/fondled you	4724 (43.7)	3884 (35.9)	2283 (35.9)	2014 (31.6)
Someone had you try to arouse them	3765 (34.9)	3100 (28.8)	2297 (36.1)	2062 (32.4)
Someone rubbed their genitals against you in sexual way	3436 (32.0)	2884 (26.9)	1587 (25.0)	1446 (22.8)
Sexual intercourse	2157 (20.0)	1721 (15.9)	1321 (20.8)	1138 (17.9)
Someone tried to put their penis in your mouth	913 (8.5)	643 (6.0)	74 (1.2)	43 (0.7)

Table 6a– Table 6g give the frequencies ( **for Mothers only**) for each of the seven experiences along with whether the mother had wanted the event to have occurred with that particular perpetrator. Note that only those answering the sub-question on whether they wanted it to happen or not are reported here. For example, in Table 6a it can be seen that the two most common perpetrators to flash at the mother were boyfriend (3.1%, of which 67.5% of mothers wanted this to happen) and stranger (15.8%, of which 98.2% did not want this to happen). 58 mothers (0.5%) had had sexual intercourse with a stranger prior to age 16, of which 81.5% had not wanted this to happen; and 100 mothers reported having intercourse with a parent/parent-figure, sibling or another relative (unwanted event with that person 93.2%, 81.0 % and 97.1% respectively) ( Table 6f).

**Table 6a.** Perpetrator of sexual exposure to the mother prior to age 16 (n=10,977).

Perpetrator	N (%) with this history	Unwanted * N (%)	Not sure * N (%)	Wanted * N (%)
Boyfriend	389 (3.1)	59 (15.5)	65 (17.1)	257 (67.5)
Girlfriend	59 (0.5)	24 (43.6)	16 (29.1)	15 (27.3)
Parent/figure	124 (1.0)	109 (88.6)	8 (6.5)	6 (4.9)
Sibling	135 (1.1)	85 (64.9)	34 (26.0)	12 (9.2)
Other relative	170 (1.4)	148 (88.6)	16 (9.6)	<5
Family friend	167 (1.3)	150 (91.5)	11 (6.7)	<5
Stranger	1968 (15.8)	1834 (98.2)	28 (1.5)	5 (0.3)
Other person	157 (1.3)	119 (82.1)	22 (15.2)	4 (2.8)

*Omitted are those not answering the ‘wantedness’ sub-question

**Table 6b.** Perpetrator of masturbation in front of mother prior to age 16 (n=10,977) (<5 may contain zero).

Perpetrator	N (%) with this history	Unwanted * N (%)	Not sure * N (%)	Wanted * N (%)
Boyfriend	316 (2.9)	26 (8.4)	73 (23.6)	210 (68.0)
Girlfriend	26 (0.2)	5 (19.2)	5 (19.2)	16 (61.5)
Parent/figure	61 (0.6)	57 (96.6)	<5	<5
Sibling	59 (0.5)	39 (66.1)	19 (32.2)	<5
Other relative	59 (0.5)	54 (93.1)	<5	<5
Family friend	80 (0.7)	75 (93.8)	<5	<5
Stranger	321 (2.9)	293 (98.0)	6 (2.0)	<5
Other person	57 (0.5)	43 (82.7)	8 (15.4)	<5

*Omitted are those not answering the ‘wantedness’ sub-question

**Table 6c.** Perpetrator of touched or fondled mother (including breast or genitals or attempted to arose her sexually) prior to age 16 (n=10,977) (<5 may contain zero).

Perpetrator	N (%) with this history	Unwanted * N (%)	Not sure * N (%)	Wanted * N (%)
Boyfriend	3811 (34.7)	144 (4.2)	787 (21.1)	2789 (74.8)
Girlfriend	111 (1.0)	17 (15.3)	40 (36.0)	54 (48.6)
Parent/figure	186 (1.7)	175 (96.2)	6 (3.3)	<5
Sibling	144 (1.3)	100 (70.4)	25 (17.6)	17 (12.0)
Other relative	246 (2.2)	200 (84.0)	30 (12.6)	8 (3.4)
Family friend	280 (2.6)	235 (86.4)	30 (11.0)	7 (2.6)
Stranger	219 (2.0)	204 (95.8)	6 (2.8)	<5
Other person	161 (1.5)	111 (77.6)	25 (17.5)	7 (4.9)

*Omitted are those not answering the ‘wantedness’ sub-question

**Table 6d.** Perpetrator who tried to have the mother arouse them or touch their body sexually prior to age 16 (n=10,977) (<5 may contain zero).

Perpetrator	N (%) with this history	Unwanted * N (%)	Not sure * N (%)	Wanted * N (%)
Boyfriend	3166 (28.8)	244 (7.9)	646 (21.0)	2191 (71.1)
Girlfriend	78 (0.7)	12 (15.6)	19 (24.7)	46 (59.7)
Parent/figure	111 (1.0)	107 (97.3)	<5	<5
Sibling	107 (1.0)	75 (71.4)	20 (19.0)	10 (9.5)
Other relative	142 (1.3)	120 (85.7)	13 (9.3)	7 (5.0)
Family friend	160 (1.5)	136 (86.6)	17 (10.8)	<5
Stranger	104 (0.9)	92 (92.0)	6 (6.0)	<5
Other person	92 (0.8)	64 (80.0)	10 (12.5)	6 (7.5)

*Omitted are those not answering the ‘wantedness’ sub-question

**Table 6e.** Perpetrator who rubbed their genitals against the mother in a sexual way prior to age 16 (n=10,977) (<5 may contain zero).

Perpetrator	N (%) with this history	Unwanted * N (%)	Not sure * N (%)	Wanted * N (%)
Boyfriend	2895 (26.4)	156 (5.6)	612 (21.8)	2035 (72.6)
Girlfriend	24 (0.2)	<5	8 (36.4)	13 (59.1)
Parent/figure	100 (0.9)	95 (96.9)	<5	<5
Sibling	73 (0.7)	55 (76.4)	12 (16.7)	5 (6.9)
Other relative	121 (1.1)	110 (91.7)	8 (6.7)	<5
Family friend	129 (1.2)	108 (87.1)	12 (9.7)	<5
Stranger	133 (1.2)	118 (91.5)	8 (6.2)	<5
Other person	105 (1.0)	67 (73.6)	18 (19.8)	6 (6.6)

*Omitted are those not answering the ‘wantedness’ sub-question

**Table 6f.** Perpetrator who had sexual intercourse with the mother prior to age 16 (n=10,977) (<5 may contain zero).

Perpetrator	N (%) with this history	Unwanted * N (%)	Not sure * N (%)	Wanted * N (%)
Boyfriend	1963 (17.9)	57 (3.0)	275 (14.5)	1559 (82.4)
Girlfriend	N/A	N/A	N/A	N/A
Parent/figure	44 (0.4)	41 (93.2)	<5	<5
Sibling	21 (0.2)	17 (81.0)	<5	<5
Other relative	35 (0.3)	34 (97.1)	0	<5
Family friend	47 (0.4)	37 (8.7)	5 (10.9)	<5
Stranger	58 (0.5)	44 (81.5)	5 (9.3)	5 (9.3)
Other person	31 (0.3)	20 (71.4)	5 (17.9)	<5

*Omitted are those not answering the ‘wantedness’ sub-question

**Table 6g.** Perpetrator who tried to put penis in mother’s prior to age 16 (n=10,977) (<5 may contain zero).

Perpetrator	N (%) with this history	Unwanted * N (%)	Not sure * N (%)	Wanted * N (%)
Boyfriend	757 (6.9)	151 (20.6)	153 (20.9)	429 (58.5)
Girlfriend	N/A	N/A	N/A	N/A
Parent/figure	42 (0.4)	40 (100.0)	<5	<5
Sibling	18 (0.2)	17 (94.4)	<5	<5
Other relative	33 (0.3)	30 (93.8)	<5	<5
Family friend	37 (0.3)	34 (94.4)	<5	<5
Stranger	25 (0.2)	22 (91.7)	<5	<5
Other person	25 (0.2)	19 (86.4)	<5	<5

*Omitted are those not answering the ‘wantedness’ sub-question

From these questions a derived definition of abuse was obtained (see Table 7). Abuse was defined as any actions by a stranger regardless of whether the respondent stated they wanted this to happen or not; events instigated by a boy/girlfriend only if the respondent said this was unwanted; and any event where a parent/parent figure, sibling, other relatives, family friend or other person was involved. Therefore, for each of these seven questions, a variable was derived distinguishing each event by a stranger from an unwanted or abusive event by a non-stranger. For example, mother being flashed at (c830-c846), using the following rules:

**Table 7.** Frequencies Mother (c) and Partner (pd) derived variables (DV) indicating unwanted or abusive action by a non-stranger vs. action by a stranger prior to the age of 17.

	Mothers			Partners		
Event/variable name	Did not happen	Stranger	Non-Stranger	Did not happen	Stranger	Non-stranger
Someone purposely flashed/ exposed themselves to you (c/pd848)	8660 (77.9)	1874 (16.9)	585 (4.7)	6496 (94.7)	234 (3.4)	130 (1.9)
Someone masturbated in front of you (c/pd868)	10507 (95.9)	198 (1.8)	246 (2.2)	6633 (96.7)	69 (1.0)	159 (2.3)
Someone touched/fondled you (c/pd888)	10006 (90.0)	189 (1.7)	924 (8.3)	6589 (96.1)	94 (1.4)	175 (2.6)
Someone had you try to arouse them (c/pd908)	10312 (92.7)	87 (0.8)	720 (6.5)	6624 (96.6)	65 (0.9)	170 (2.5)
Someone rubbed their genitals against you in sexual way (c/pd928)	10464 (94.1)	117 (1.1)	538 (4.8)	6745 (98.3)	32 (0.5)	83 (1.2)
Sexual intercourse (c/pd948)	10878 (97.8)	47 (0.4)	194 (1.7)	6804 (99.2)	24 (0.3)	33 (0.5)
Someone tried to put their penis in your mouth (c/pd968)	10626 (98.1)	15 (0.1)	192 (1.8)	6814 (99.3)	13 (0.2)	34 (0.5)
**DV: Any sexual abuse**	**7861 (70.7)**	**1736 (15.6)**	**1522 (13.7)**	**6177 (90.1)**	**348 (5.1)**	**330 (4.8)**

If c843=2 then c848=1 (stranger).

If (c832=1 or c834=1 or c835=2 or c837=2 or c839=2 or c841=2) then c848=2 other abusive flashing; Otherwise c848=3. These gave the value labels for the derived variable (DV) c848 as stranger, non-stranger, did not happen and missing. These derived variables are indicated in the *Extended data* (Supplementary Table 1 ( Iles-Caven, 2024)) by the prefix DV and suffixed by an *.

Table 7 gives the derived variable frequencies for both mothers and partners for all seven experiences. Total mother stranger abuse incidence was 15.6% compared with 13.7% for non-stranger abuse. For partners the figures were 5.1% and 4.8% respectively.

The final question in this section asked the age at the time of the first occurrence with the range 1–20 years (although the question stipulated <16 years). Table 8 groups the ages for both parents at <5, 6–11 and 12+ years, with most experiences occurring in the 12+ age group.

**Table 8.** Age at which the sexual experience first occurred.

Event	Mothers			Partners		
	≤5 yrs	6–11 yrs	12+ yrs	≤5 yrs	6–11 yrs	12+ yrs
Someone purposely flashed/ exposed themselves to you	120 (4.6)	1021 (39.3)	1454 (56.0)	26 (2.5)	260 (40.3)	368 (57.1)
*Valid cases / variable name*	*2595*	*c847*		*644*	*pd847*	
Someone masturbated in front of you	37 (4.5)	276 (33.7)	506 (61.8)	<5	141 (27.6)	366 (71.6)
*Valid cases / variable name*	*819*	*c867*		*511*	*pd867*	
Someone touched/fondled you	79 (1.9)	525 (12.7)	3533 (85.4)	6 (0.3)	214 (10.8)	1754 (88.6)
*Valid cases / variable name*	*4137*	*c887*		*1974*	*pd887*	
Someone had you try to arouse them	51 (1.6)	345 (10.7)	2814 (87.7)	6 (0.3)	196 (10.1)	1745 (89.6)
*Valid cases / variable name*	*3210*	*c907*		*1947*	*pd907*	
Someone rubbed their genitals against you in sexual way	44 (1.5)	261 (9.0)	2582 (89.4)	<5	103 (7.8)	1214 (92.0)
*Valid cases / variable name*	*2887*	*c927*		*1319*	*pd927*	
Sexual intercourse	13 (0.7)	68 (3.6)	1816 (95.7)	<5	26 (2.4)	1070 (97.5)
*Valid cases / variable name*	*1897*	*c947*		*1097*	*pd947*	
Someone tried to put their penis in your mouth	19 (2.3)	71 (8.7)	729 (89.0)	6 (8.2)	25 (34.2)	42 (57.5)
*Valid cases / variable name*	*819*	*c965*		*73*	*pd965*	

When the index children (G1s) were aged 7 years, the mother and partner were asked to describe their sexual orientation, the sex of their partners and live-in partners since the child was born and their current live-in partner. Frequencies are given in Table 9.

**Table 9.** Mother’s and Partner’s sexual orientation and gender of partners at 7 years after the birth of the index child. (<5 may contain zero).

	Mothers	Partners
*Sex of partners respondent * *had since child born*	*m3200*	*pk3200*
Only male	7835 (97.6)	48 (1.2)
Mostly male	28 (0.3)	<5
Both male and female	19 (0.2)	7 (0.2)
Mostly female	<5	31 (0.8)
Only female	31 (0.4)	3720 (96.6)
No partner	113 (1.4)	45 (1.2)
	8030	3852
*Respondent’s description* * of their sexual orientation*	*m3201*	*pk3201*
Heterosexual	7699 (99.1)	3855 (99.4)
Bisexual	54 (0.7)	19 (0.5)
Lesbian/homosexual	15 (0.2)	6 (0.2)
	7768	3880
*Respondent currently living* * with a partner*	*m3202*	*pk3202*
Yes, male partner	7188 (88.8)	30 (0.8)
Yes, female partner	12 (0.1)	3817 (97.7)
Yes, multiple partners	<5	<5
Not living with a partner	897 (11.1)	58 (1.5)
	8098	3907
*Sex of partners has lived* * with since birth*	*m3202*	*pk3203*
Male partner(s) only	7586 (94.9)	30 (0.8)
Male and female	9 (0.1)	<5
Female partner(s) only	16 (0.2)	3801 (98.1)
Not lived with a partner	385 (4.8)	41 (1.1)
	7996	3876

## Observations/Discussion

The over-arching aim of this data note is to describe all the data collected over time from the ALSPAC parental cohorts relating to lifetime sexual experiences, sexual functioning, sexual orientation and regret. Similar information for the offspring cohort (G1s) is described in a companion data note ( Iles-Caven & Golding, 2024).

Many publications using ALSPAC data have concentrated on abuse to the offspring and/or considered all areas of ‘childhood adversity’ including sexual, physical or emotional abuse, family adversity, financial instability among others, and various outcomes (see Iles-Caven & Golding, 2024). Research has been undertaken looking at adverse childhood events (ACEs) in the mothers and subsequent outcomes in their offspring. For example, Collishaw and colleagues (2007) found that maternal childhood abuse was associated with worse behavioural trajectories between the ages of 4 and 7 years. These children were more likely to experience a range of negative life events between ages 4 and 7 years, including changes in family composition, separations from parents, “shocks and frights” and physical assaults.

A paper by Magnus *et al.* (2018) found that on examining separate psychosocial adversity constructs in the mothers, sexual abuse was inversely associated with both age at menarche and at menopause. A maternal history of childhood maltreatment (including sexual abuse) was associated with internalising and externalising difficulties in their offspring ( Plant *et al.*, 2017). A history of sexual abuse in the mothers when aged 47 was strongly associated with a higher DNA methylation age of 3.41 years ( Lawn *et al.*, 2018). 

Roberts *et al.* (2004) found that child sexual abuse <13 years of age had long-term detrimental effects on adult mental health and was associated with a variety of other outcomes, including currently being in a nontraditional family type (single mother and stepfather), teenage pregnancy, parenting behaviours, and adjustment problems in their subsequent offspring.

## Strengths and limitations

Extensive data collection in ALSPAC on sexual experiences, sexual functioning, sexually transmitted infections, along with genetic and inflammation data allow for more nuanced examination.

As with all longitudinal studies, attrition is a problem. In ALSPAC this has been higher for those who experienced more adversity during enrolment and the index pregnancy (e.g. lack of social support or inadequate housing). Subsequently in the parental cohorts, attrition is due mainly to mortality, change of address (and consequent loss to follow up), as well as a reluctance to stay involved. Therefore, selection bias becomes a problem such that the current participants are those less socio-economically deprived, and with higher educational attainments compared with those no longer taking part. However, analyses of the ways in which such attrition may bias results has been shown to be minimal ( Morgan *et al.*, 2022).

All sexual experiences data was self-reported and in the case of the G0s’ early experiences, retrospectively. For the older mothers and their partners, the events may have occurred long ago and therefore some events may have been forgotten or misremembered. If the event had been very traumatic, it may be that the respondent mentally blocked it out; conversely, less traumatic events may have been forgotten.

## Conclusion

This rich data resource has plenty to offer researchers examining the effects of early sexual experiences (particularly unwanted events/abuse) on future mental health and well-being, quality of intimate relationships, IPV (intimate partner violence), parenting styles, etc. In particular, the opportunity to examine the differences in experiences and outcomes across generations is, as yet, untapped.

## Ethics and consent

Ethical approval for the ALSPAC study was obtained from the ALSPAC Ethics and Law Committee (ALEC; IRB00003312) and the Local Research Ethics Committees. Detailed information on the ways in which confidentiality of the cohort is maintained may be found on the study website: http://www.bristol.ac.uk/alspac/researchers/research-ethics/. All methods were performed in accordance with the relevant guidelines and regulations. Informed consent for the use of data collected via questionnaires and clinics was assumed from participants following the recommendations of the ALSPAC Ethics and Law Committee at the time ( Birmingham, 2018).

## Data Availability

ALSPAC data access is through a system of managed open access. The steps below highlight how to apply for access to the data included in this paper and all other ALSPAC data. Note that variable names are included in the tables within this paper. Please read the ALSPAC access policy ( http://www.bristol.ac.uk/media-library/sites/alspac/documents/researchers/data-access/ALSPAC_Access_Policy.pdf) which describes the process of accessing the data and biological samples in detail, and outlines the costs associated with doing so. You may also find it useful to browse our fully searchable research proposals database ( https://proposals.epi.bristol.ac.uk/), which lists all research projects that have been approved since April 2011. Please submit your research proposal ( https://proposals.epi.bristol.ac.uk/) for consideration by the ALSPAC Executive Committee using the online process. You will receive a response within 10 working days to advise you whether your proposal has been approved. If you have any questions about accessing data, please email: alspac-data@bristol.ac.uk (data) or bbl-info@bristol.ac.uk (samples). The ALSPAC data management plan ( http://www.bristol.ac.uk/media-library/sites/alspac/documents/researchers/data-access/alspac-data-management-plan.pdf) describes in detail the policy regarding data sharing, which is through a system of managed open access. Open Science Framework: Extended data. https://doi.org/10.17605/OSF.IO/M3DVJ ( Iles-Caven, 2024). Data are available under the terms of the Creative Commons Attribution 4.0 International license (CC-BY 4.0).
