# Supplementary material for: Sexual experiences and behaviours of the parents in the Avon Longitudinal Study of Parents and Childhood (ALSPAC)
Source: Wellcome Open Res. 2024 Nov 6;9:240. Originally published 2024 May 9. [Version 2] doi: 10.12688/wellcomeopenres.21263.2 (PMC11574334; doi:10.12688/wellcomeopenres.21263.2)
Supplement: Peer review history

# Reviewer report: Reviewer response for version 2

**DOI:** 10.21956/wellcomeopenres.25777.r110328

**Published:** 2024-11-18

**Authors:** Ruth Lewis, Billie Turner

**Affiliations:** 1 University of Glasgow, Glasgow, UK

Thanks to authors for their careful consideration of our suggestions, and subsequent amendments. We have no further comments.

Are sufficient details of methods and materials provided to allow replication by others?

Yes

Is the rationale for creating the dataset(s) clearly described?

Yes

Are the datasets clearly presented in a useable and accessible format?

Yes

Are the protocols appropriate and is the work technically sound?

Yes

Reviewer Expertise:

NA

We confirm that we have read this submission and believe that we have an appropriate level of expertise to confirm that it is of an acceptable scientific standard.

# Reviewer report: Reviewer response for version 2

**DOI:** 10.21956/wellcomeopenres.25777.r110327

**Published:** 2024-11-07

**Authors:** Ron Roberts

**Affiliations:** 1 Kingston University, London, England, UK

No further comments

Are sufficient details of methods and materials provided to allow replication by others?

Yes

Is the rationale for creating the dataset(s) clearly described?

Yes

Are the datasets clearly presented in a useable and accessible format?

Yes

Are the protocols appropriate and is the work technically sound?

Yes

Reviewer Expertise:

I was the lead author on some of the work cited in this paper (Roberts et al., 2004), with my then interest being on the intergenerational effects of CSA.

I confirm that I have read this submission and believe that I have an appropriate level of expertise to confirm that it is of an acceptable scientific standard.

# Reviewer report: Reviewer response for version 1

**DOI:** 10.21956/wellcomeopenres.23517.r99692

**Published:** 2024-10-25

**Authors:** Ruth Lewis, Billie Turner

**Affiliations:** 1 University of Glasgow, Glasgow, UK

This is a clearly presented, comprehensive and valuable dataset describing the sexual experiences and function of mothers and their partners who took part in the Avon Longitudinal Study of Parents and Children (ALSPAC). This dataset provides valuable opportunity for comparison with other survey data, enabling researchers to examine important questions (e.g. relating to sexual experiences and function among parents, and intergenerational impacts of parents’ childhood experiences on their children’s health).  

 Our suggestions for improvement relate to some lack of clarity or inconsistencies in the terminology used to describe the different constructs and variables within the dataset, which could have implications for data discoverability and comparability. Below we provide some specific comments and suggestions for the authors to consider:  

1. In the abstract, it would be useful to clarify that there are also data available on parents’ experiences of sexual assault as an adult.  
2. The title and introduction refer to data on “sexual attitudes”, but it is not clear which attitudinal data this refers to as the tables report on sexual experience, satisfaction, functioning, and sexual orientation.  
3. We suggest a clearer characterisation of the relational dimensions of mother-partner relationships for which data on satisfaction are available. For instance, what is meant by “satisfaction with […] non-sexual sides of their relationships” (p1, p10 table)? Does this refer to parents’ reports of satisfaction with affection? Moreover, on page 10, it is noted that “Over half the mothers and partners reported being very satisfied with their (non-sexual) relationship in general”, which suggests a completely non-sexual relationship rather than a “non-sexual side” of the relationship.  Could this be clarified? 
4. The methods section (p4) refers to “sexual identity” whereas in the rest of the paper this is referred to as “sexual orientation”. These terms are typically conceptualised as distinct, and it would be valuable to be consistent with regards description of the construct that was measured. 
5. It would be helpful to clarify the definitions of sexual drive and desire within ALSPAC. These are sometimes referred to in the data note separately, and sometimes as a hybrid term (“sexual drive/desire”, p8, p10). Is desire a relationship-specific measure of desire to have sex with their partner, or a measure of individual interest in sex (not partner specific)? 
6. P.17 mentions data relating to “regret”, but it is not clear which data this refers to, and regret is not described as a variable in any of the tables. 

Are sufficient details of methods and materials provided to allow replication by others?

Yes

Is the rationale for creating the dataset(s) clearly described?

Yes

Are the datasets clearly presented in a useable and accessible format?

Yes

Are the protocols appropriate and is the work technically sound?

Yes

Reviewer Expertise:

NA

We confirm that we have read this submission and believe that we have an appropriate level of expertise to confirm that it is of an acceptable scientific standard, however we have significant reservations, as outlined above.

# Response

**Published:** 2024-11-04

**Authors:** Yasmin Iles-Caven

**Affiliations:** Bristol Medical School: Population Health Sciences, University of Bristol, Bristol, UK

In the abstract, it would be useful to clarify that there are also data available on parents’ experiences of sexual assault as an adult.  

**Response:** **Many thanks for your useful comments on our paper.** **We have clarified the abstract as you suggest:  **“ **During the index pregnancy the mothers were asked (at 8 months post-delivery for partners) about their early sexual experiences to identify sexual abuse, the age at which an event first occurred, along with the type of perpetrator (e.g. stranger, relatives, boy/girlfriend). There are also data on parents’ experiences of sexual assault as an adult.”**

 The title and introduction refer to data on “sexual attitudes”, but it is not clear which attitudinal data this refers to as the tables report on sexual experience, satisfaction, functioning, and sexual orientation.  

**Response: We agree and have altered the title, abstract and introduction accordingly. The sexual attitudes data are reported in a soon-to-be published paper: Iles-Caven Y, Golding J, Joinson C, Fraser A, Northstone K.  Sexual attitudes, enjoyment and regret in the parent and offspring generations of the Avon Longitudinal Study of Parents and Childhood (ALSPAC): 2022 data sweeps. Wellcome Open Res 2024; 23059).**

 We suggest a clearer characterisation of the relational dimensions of mother-partner relationships for which data on satisfaction are available. For instance, what is meant by “satisfaction with […] non-sexual sides of their relationships” (p1, p10 table)? Does this refer to parents’ reports of satisfaction with affection? Moreover, on page 10, it is noted that “Over half the mothers and partners reported being very satisfied with their (non-sexual) relationship in general”, which suggests a completely non-sexual relationship rather than a “non-sexual side” of the relationship.  Could this be clarified? 

**Response:** **This has been clarified in Table 10 and the text on page 10.  The wording of these questions is clear within the context of the questionnaire:  “How satisfied are you with your overall sex life?” Followed by “How satisfied are you with your general (non-sexual) relationship with your partner?” However, the phrasing within Table 10 (to cut down the wording) was ambiguous. Similarly, we agree that “general (non-sexual) relationship” could encompass any and all facets of an intimate relationship including affection and warmth, respect, compatibility, general happiness etc and is subjective.**

****

 The methods section (p4) refers to “sexual identity” whereas in the rest of the paper this is referred to as “sexual orientation”. These terms are typically conceptualised as distinct, and it would be valuable to be consistent with regards description of the construct that was measured. 

**Response: The wording of the question asked: “How would you describe your sexuality?”.  We have changed ‘identity’ to ‘orientation’ on page 4 and changed Table 9 ‘orientation’ to ‘sexuality’.** **The text describing Table 9 (p15) now contains the wording of the question so as to be absolutely clear what was asked.**

 It would be helpful to clarify the definitions of sexual drive and desire within ALSPAC. These are sometimes referred to in the data note separately, and sometimes as a hybrid term (“sexual drive/desire”, p8, p10). Is desire a relationship-specific measure of desire to have sex with their partner, or a measure of individual interest in sex (not partner specific)? 

**Response:** **It would be difficult to separate sexual drive and sexual desire within ALSPAC as the wording in the questionnaires is “Are you worried or distressed by your current level of sex drive/desire?”  We have therefore ensured that all mentions use the hybrid term.**

 P.17 mentions data relating to “regret”, but it is not clear which data this refers to, and regret is not described as a variable in any of the tables. 

**Response: Thank you for pointing out this error. It has been corrected. The sexual regret data was collected in 2022 and will be published shortly (see ref above under Q2).**

# Reviewer report: Reviewer response for version 1

**DOI:** 10.21956/wellcomeopenres.23517.r87486

**Published:** 2024-07-30

**Authors:** Ron Roberts

**Affiliations:** 1 Kingston University, London, England, UK

This is an extremely important paper laying out the wealth of data on the sexual experiences, behaviours and attitudes in the parents who have participated in ALSPAC. To my knowledge this is the most extensive set of such data, anywhere in the world. As the authors note, and I wholeheartedly agree - this data has enormous potential for researchers - in particular, in relation to studying the similarities and differences in experiences and outcomes across generations. Though researchers interested in sexual behaviour per se will find plenty to interest them.

 With the additional data the authors have collected, there is the opportunity to clarify the way in which early abuse and adverse circumstances trigger subsequent epigenetic changes.  I think the data also offers further opportunities to disentangle putative genetic effects on behaviour from environmental effects, an issue of continuing importance – not just theoretically but also potentially practically in the field of interventions.

 On a minor note – I found that some of the data in table. would benefit from clarification. For example, it is stated that 5.1% of mothers self-reported being sexually abused before 17 years of age, whilst the same table gives a figure of 4.1% of sexually assaulted ever.  Sexual abuse and sexual assault are not identical terminology and may well mean different things for some participants, and I think some small comment to this effect and what this signifies would be helpful to the reader.

Are sufficient details of methods and materials provided to allow replication by others?

Yes

Is the rationale for creating the dataset(s) clearly described?

Yes

Are the datasets clearly presented in a useable and accessible format?

Yes

Are the protocols appropriate and is the work technically sound?

Yes

Reviewer Expertise:

I was the lead author on some of the work cited in this paper (Roberts et al., 2004), with my then interest being on the intergenerational effects of CSA.

I confirm that I have read this submission and believe that I have an appropriate level of expertise to confirm that it is of an acceptable scientific standard.

# Response

**Published:** 2024-11-04

**Authors:** Yasmin Iles-Caven

**Affiliations:** Bristol Medical School: Population Health Sciences, University of Bristol, Bristol, UK

Very many thanks for your review.  In response to your comment below:

 On a minor note – I found that some of the data in table 4. would benefit from clarification. For example, it is stated that 5.1% of mothers self-reported being sexually abused before 17 years of age, whilst the same table gives a figure of 4.1% of sexually assaulted ever.  Sexual abuse and sexual assault are not identical terminology and may well mean different things for some participants, and I think some small comment to this effect and what this signifies would be helpful to the reader.

 The battery of life events questions were asked antenatally (cfiles/pbfiles) comprising a 31-item list of generally negative events (A parent died, A sibling died, Your parents divorced, were seriously ill etc) that occurred in childhood (<17 years) with the degree of effect on the respondent (affected me a lot etc). One item was ‘You were sexually abused’ (5.1%). A series of questions in another antenatal questionnaire (dfiles/pafiles) asked about various events that may have occurred within their life so far (had a bicycle accident, road traffic accident, nearly drowned, injured in a fight etc and ‘You were sexually assaulted’ 4.1%) that had ever occurred; whether they had been hospitalised, consulted their GP, or treated at home and the age at which this event occurred.  It is conceivable that those who ticked abuse may not have ticked assaulted.  This may be because they very likely mean different things to different people. For instance, ‘sexual abuse’ may be considered something that happens to children (or other vulnerable people) and possibly by a perpetrator who is a relative, teacher or an adult known to the child. Some may regard ‘sexual assault’ as something that happens to anyone post-puberty and may include anything from rape to inappropriate touching, particularly by a stranger.  

 We have added a few lines to the Limitations section about this.
